# Book Review

**Published:** 1909-09

**Authors:** 


**Green's Encyclopedia and Dictionary of Medicine and Surgery.** Vol. X. Thiersch—Zymotic. Pp. xii, 609. Edinburgh: Wm. Green & Sons.—This concluding volume completes the series. The promises made in the Introduction have been accomplished. A medical dictionary has been incorporated with the *Encyclopedia Medica*, as almost all the articles which originally appeared still remain, but incorporated with a large number of new ones. An elaborate system of cross references adds very much to the value of the work. Important advances in medical and surgical practice have been noticed, either in the form of new articles or as additions to the former ones, and additional illustrations have been supplied. A supplementary volume is in active preparation, and will appear in 1909. This quinquennium of medicine and surgery is expected to be a trustworthy guide to the really important additions which the past five years have made to our means of combating disease, deformity and death.

**The Future of Medicine.** By SYDNEY W. MACILWAIN. Pp. vi, 44. London: P. S. King & Son. 1908.—The author holds that the term disease is persistently and officially misused, that the pathological theory of disease is false, that medicine has always been dominated and vitiated by a false interpretation of Nature, and that henceforth the specific word "disease" must only be applied to definite conceptions that imply the correlation of cause and effect. It is consoling to find that the surgeon does not, as a rule, mislead himself with false diagnosis. The physician is the pathologist, and the ideal of the pathologist is directly opposed to the clinical correlation of cause and effect; however, a knowledge of bacteriology has completed a revolution in our conception of the nature of specific diseases, and even the misguided physician may now reach a true interpretation of Nature by adopting conceptions that imply the correlation of cause and effect.

**Medico-Chirurgical Transactions.** Vol. XC (and last). London: Longmans, Green and Co. 1907.—This series of annual *Transactions* commenced in 1805, and has now concluded, being replaced by the *Proceedings of the Royal Society of Medicine*. The President, at the last annual meeting, remarked that "although this may be the last annual meeting of the Royal Medical and Chirurgical Society as such, we shall hope that, strengthened by our association with its other constituents, widened by the incorporation of bodies of diverse interests, and invigorated by the infusion of fresh energy, we shall add to the caution and experience of age the enthusiasm and experience of youth, and take a part in the advancement of our art not unworthy of the record of our Society during the past hundred years." The ninety volumes will form the best possible memorial of the Society's life.

**Transactions of the Association of American Physicians.** Vol. XXIII. Philadelphia: Printed for the Association. 1908.—This volume forms the report of the Proceedings of the Twenty-Third Annual Meeting of the Association, held in Washington, D.C., May 12th and 13th, 1908. It is impossible to attempt more than a brief review of some of the leading features of the communications published. In the presidential address reference is made to the loss the Association has sustained during the year by the untimely death of Dr. James Carroll, who in his own person permitted the crucial experiment to be performed which proved the transmission of yellow fever by mosquitos. Born in England in 1854, an emigrant to Canada at fifteen years of age, a private soldier in the United States army at twenty, he rose from the latter position to that of a major and surgeon in the medical corps. An aptitude for bacteriology, and skill in experiment, led ultimately to his inclusion in the Commission appointed in 1900 to search out the cause of yellow fever. "The part taken by Carroll in permitting himself to be bitten by the infected mosquito, the infliction of the deadly disease, his desperate illness, and his death seven years later from its remote effects, constitute the acts in a tragedy scarcely equalled in the intensity of its action and the heroism of its *dramatis persona princeps*." Among the papers, Baldwin's "Study of Opsonic Phenomena" deals with some of the divergencies between theory and practice in opsonic estimation. Emphasis is laid upon the limitations of the phenomena of the opsonins as a measure of specific resistance and in diagnosis, "limitations which are more apparent as additional facts have been brought out of clinical studies." The results of six or seven daily opsonic estimations, before and after the subcutaneous tuberculin test for diagnosis, "do not encourage us to expect much practical use of the index in pulmonary cases in the usual method of its application." There is an interesting summary of 400 cases of epidemic meningitis treated with the anti-meningitis serum. contributed by Flexner and Jobling, in which the beneficial influence of the early injection is rendered sufficiently obvious by the following table:—

| Period of Injection. | Cases. | Recovered. | Died. | |
|---|---|---|---|---|
| First to third day .. .. | 123 | 107 | 16 | 16.5 per cent. |
| Fourth to seventh day .. | 126 | 96 | 30 | 23.8 per cent. |
| Later than seventh day .. | 112 | 73 | 39 | 35 per cent. |

A paper on "Ligation of the Coronary Arteries" (Miller and Matthews) embodies some experimental work which throws considerable light on angina pectoris and the drugs employed for its relief. The most striking conclusion arrived at is that in attacks of angina accompanied by high blood-pressure nitroglycerine produces relief, but with a low blood-pressure (" Leyden has shown that the majority of patients suffering from angina do not have hypertension. In patients with hypertension, if the attack is severe, a very marked fall in pressure has occurred.") cardiac stimulants, such as digitalis and caffeine, are indicated. Meyer, on " The Relation of the Auditory Centre to Aphasia," demonstrates the definite localisation of the sensory side of aphasia, a paper which was received by one of his critics " as one of the most valuable contributions we have had in recent years on the subject." Herter, in his " Infantilism from Intestinal Infection," gives details of the most careful investigations of the metabolism in such cases, and holds out hope of even re-establishing the processes of growth by rational therapeutic interference in chronic intestinal infantilism. The whole volume, however, is full of excellent work, and the transactions of such a meeting as this embody the best results of a year's progress in medicine, as seen by our eminent American colleagues.

**Transactions of the Medico-Chirurgical Society of Edinburgh.** Vol. XXVII. New Series. Edinburgh: James Thin. 1908.—We wish to give this " New Exchange " a cordial welcome. The record of the work done during the session 1907—1908 shows itself to be a valuable contribution to medical literature, and a useful reminder of many most interesting meetings.

**St. Bartholomew's Hospital Reports.** Vol. XLIV. London: Smith, Elder & Co. 1909.—This volume contains a fair proportion of contributions by members of the hospital staff, on which we congratulate the editors, and a welcome absence of mediocre theses which have served to satisfy the examiners at Oxford or Cambridge, but have not always contributed to the " Commonwealth of letters." Dr. Herringham gives the results of examination of the *post-mortem* records for all cases of gastric or duodenal ulcer, and finds that gastric or duodenal ulcer occurred in 2 per cent. of all bodies examined in twenty years (10,161 necropsies), and was directly fatal in 1.2 per cent. of them. He goes on to consider gastro-stasis in the light of *post-mortem* examination, and finds six fatal cases of hæmatemesis which might properly be regarded as due to this condition, no ulcer or erosion being present. He also quotes eight cases operated on for this symptom. Dr. Tooth has a most interesting account of a case of tumour of frontal lobe and corpus callosum in a woman of 33, which presented symptoms suggestive of a cerebellar lesion. The mental condition was, as he thinks, characteristic of frontal tumour, especially in the feature of " irrelevant jocularity, even hilarity (*witzelsucht*—' jocular mania '), which, considering the sad plight of such patients, is peculiarly pathetic." Another feature was the presence of apraxia or dyspraxia, an inability to perform certain familiar purposive movements without motor or sensory paralysis or ataxia, of which he says "there is probably a future for this symptom as a means of diagnosis." Dr. Lewis Jones describes the new electrical department and its work, showing the increasing importance of electro-therapeutics in diagnosis and treatment. Dr. Sheffield Neave's account of sleeping sickness is one of the best monographs on the subject we have read. Mr. D'Arcy Power, who inherits or has acquired an unequalled gift for clinical word-painting, writes of some cases illustrating the surgery of the spleen. The cases were briefly: (1) Removal of an enlarged and displaced spleen (probably syphilitic hypertrophy)—patient alive and well nine years afterwards; (2) removal of the spleen for primary sarcoma—death six months later; and three cases of laceration of the spleen, with two recoveries. Probably an unusually large series of successful operations on an organ seldom coming under the surgeon's notice. Mr. Sydney Scott publishes some observations on the "Histology of the Human Labyrinth in Meningitis," which we think have already appeared in the *Transactions* of the Royal Society of Medicine, although perhaps not in the same form. Mr. R. C. Elmslie contributes a paper on "Osteitis Deformans, with a report upon two cases in which sarcoma of one of the affected bones arose." An excellent paper with a still more excellent bibliography. From China Dr. J. L. Maxwell reports six cases of chronic intussusception—excision was practised in five cases, right enterostomy in one; one case recovered. The youngest case was 13 years of age. To us in England the condition appears extremely rare. Dr. Halls Dally gives a detailed account of "The Diaphragm in Man: a record of our present knowledge of its development, relationships, structure and mode of action." This is based upon a thesis presented to the University of Cambridge for the M.D. degree, and is an example of the painstaking work by which the sum of our knowledge is occasionally advanced under the too little appreciated method of conferring the doctorate in medicine at the older Universities. Rarely in recent years have the St. Bartholomew's Hospital Reports contained such a collection of illuminating papers or attained such a uniformly high literary standard.

**Manual of Operative Surgery.** By H. J. Waring, M.S., M.B. Third Edition. Pp. xxxi, 750. London: Oxford Medical Publications. 1909.—This manual is one of the best of its kind, and the new edition presents many additions and improvements. The most noteworthy point of excellence is the character of the illustrations, which are profuse, artistic and accurate. It is unfortunate, however, that the book has received so much addition to the descriptive matter that it is much more than a mere student's guide to operations; and yet these additions leave it very far short of being an up-to-date practical guide to operative work. We think the author and his collaborators ought to make up their mind which it is to be—a student's guide for examinations, in which case a large amount of matter ought to be deleted, or a treatise on practical operative surgery, in which case much will have to be added, and the descriptive matter brought up to date. Many operations of an essentially similar character are described with wearisome repetition (*e.g.* the operations for the radical cure of hernia, and those for the removal of the uterine appendages), whereas from the student's point of view the essential plan of the operation ought to be given, followed by a short summary of different details, in which authorities and morbid conditions require variation. That the book cannot be regarded as an up-to-date exposition of operative methods is shown by the following omissions, for example: The use of muscle flaps in the closure of the femoral ring, the use of Michel's clips for skin suture, the relation to be observed between the direction of the gut and the stomach in anastomosis operations, the excision of strictures of the urethra, the relation of the levator ani muscles to the surgery of the female pelvic floor, the enucleation of tonsils, the employment of differential pressure in operations upon the lungs and pleura, Kocher's method of removal of the Gasserian ganglion, and the treatment of extroversion of the bladder by implantation of the ureters in the rectum. Of the special departments, that relating to the ear is described by Mr. West clearly and well, and with beautiful illustrations; that related to the eye is so meagre as to be hardly worth inclusion.

**Injuries and Diseases of the Knee-Joint.** By Sir Wm. Bennett, K.C.V.O., F.R.C.S. Pp. xv, 236. London: James Nisbet & Co., Ltd. 1909.—This book is to be heartily commended as a clinical work on the knee-joint embodying the extensive experience of the author. It is illustrated by several plates and figures, many being X-ray photographs. It would be difficult to refer to the many excellent features of the book, but we regard the chapter on acute suppuration in the knee-joint as a particularly good one; and the author says he has every reason to anticipate that, with judicious treatment, a sound limb will result. He points out that suppuration may occur without an open wound, particularly after influenza, and that in some forms aspiration may be as effective as incision in treatment. For relief of pain in the knee he favours heat rather than cold. One chapter is devoted to painless effusion occurring in women and young girls, and in boys at the adolescent period, which may be cured when one recognises the association with a genital cause. He remarks that there is no reason for suspecting tubercle as a cause of painless effusion unless there be persistent increase of local heat. He has judicious words on the question of wiring a fractured patella, and states that a death occurred in London only last year from that operation. Whatever method may be adopted for fractured patella, it is important to employ massage, and to ensure the mobility of the upper fragment. At the expense of being thought unorthodox, he disagrees with immobilisation in the early stages of tuberculous disease, reserving it for cases showing local heat, pain on standing, or persistent contraction of the ham-strings. The final chapter is upon induced hyperæmia, to which he attaches some value, although it is of decidedly more value in non-tuberculous cases and harmful in tuberculous ones if it cause pain. We have only noticed one misprint, on p. 235, and hope that an index will be added to any future edition.

**Essays on the Position of Abdominal Hysterectomy in London.** By JOHN BLAND-SUTTON, F.R.C.S. Pp. 90. London: James Nisbet & Co., Ltd. 1909.—Only the first chapter deals with the subject named in the title, the rest of the book dealing in an interesting and practical manner with such subjects as injuries of the ureters, thrombosis and embolism after operation and other matters, the most instructive of which is the chapter on adenomyoma of the uterus and tuberculosis of the endometrium. Abdominal hysterectomy does not appear to be different in London to the same operation in the provinces—at all events in Bristol. The operation is one of low mortality, and the author agrees with many of us that the supra-vaginal operation is preferable to total hysterectomy in most cases, and that the conservation of at least one ovary is desirable if the patient has not reached the menopause. On page 13, line 8, the word "fourth" should be "fortieth."

**Transactions of the American Proctologic Society, 1908.**—The present volume contains the papers and discussions of the tenth annual meeting held during 1908 of this small Society of some thirty or forty members. The subjects embrace the common affections of the rectum, amebiasis, dysentery, and the choice of an anæsthetic in rectal surgery. Colostomy is advocated as the best treatment for syphilitic stricture of the rectum. An interesting case of spontaneous ileo-sigmoidostomy from cancer is described, as well as one in which portions of a skull were removed from the rectum. Indications are given that, as a separate body, the Proctologic Society may cease to exist.

**The Diseases of Women.** By J. BLAND-SUTTON, F.R.C.S., and ARTHUR E. GILES, M.D., B.Sc. Fifth Edition. Pp. viii, 536. London: Rebman, Limited. 1906.—We look upon this volume as being for its size probably one of the best expositions of the subject so far published. It is well written, well illustrated and original. Several excellent points are made in various chapters, which we think should be more generally emphasised; for example, the relations between the appendix and inflammatory affections of the right uterine appendages, although on. In this case proper emphasis is given to the necessity of examining the appendix in such cases and if necessary removing it, while the possibility of this structure being damaged in the course of the removal of tissues is clearly demonstrated. As we should expect, the volume contains some of the latest observations on tubal pregnancy; the chapters on this subject should be widely read by practitioners. Some interesting information is given as to the forms of degeneration in uterine by no means unusual, is not in our experience sufficiently insisted fibroids, but the authors, while rightly insisting on the originally semi-malignant (sarcomatous) nature of some myxomatous tumours, are rather sceptical as to the occurrence of sarcomatous changes. From our experience, necessarily more limited, we are able to substantiate the occurrence of this in cases in which fibroids, known to have existed for years, suddenly underwent marked increase in size, sarcomatous tissue was found in them, and two fatal cases metastases in the lungs. We repeat that this in volume will amply repay perusal, and look upon it as one of the most valuable recent contributions to the literature of the subject.

**Outlines of the Diseases of Women.** By JOHN PHILLIPS, M.A., M.B. Fourth Edition. Pp. xiv, 282. London: Charles Griffin & Company, Limited. 1906.—This small book will be found very valuable by senior students while engaged in the study of practical gynecology. It is an attempt to present in a condensed and abbreviated form the various methods of practice which are necessary for the student, and is not intended to take the place of the more advanced and larger manuals, although it contains within a small space a very large amount of valuable information. We consider that the author has produced a very valuable contribution to the literature of the subject, since, while up-to-date information is given, too many details are avoided. If these should be wanted it is only necessary to consult the works referred to at the end of each chapter. Some points might be improved. The anatomical description of the pelvic floor is rather sketchy, and we doubt whether it would be accepted as sufficient by the most modern anatomists. It seems rather a mistake in present-day works to introduce plates and figures of instruments of a make which do not stand boiling, and one or two old and incorrect anatomical diagrams might well have been omitted.

**Diseases of the Eye.** By CHARLES H. MAY, M.D., and CLAUD WORTH. Second Edition. Pp. viii, 400. London: Baillière, Tindall & Cox. 1908.—The second edition of this excellent hand-book has now appeared. The first edition was published in March, 1906. That a second edition is called for so soon is, we think, sufficient evidence that the book is a popular one. As the authors say, the book is intended for the student and general practitioner, and so is not overloaded with accounts of those rare conditions which one sees but once or twice in a lifetime. This second edition has been brought up to date, and several sections have been entirely rewritten. We are glad to congratulate the authors on the success of their book, and confidently recommend it to the student and practitioner for whom it is intended.

**Diseases of the Eye.** By M. Stephen Mayou. Pp. xii, 388. London: Oxford Medical Publications. 1908.—This book, which is one of the Oxford Medical Manuals, is in the unfortunate position of "falling between two stools." It is too large to be merely a cram book, and too small to make useful reading for anyone interested in the subject. For the most part it is a book of bald statements, and although the author says "it is better for the student to have a knowledge of one method or theory than a confused smattering of several," still a little explanation of "why things are so" is a great help to enable the student to remember, and to understand for himself. A great many text-books for the "unfortunate student" and busy practitioner have appeared during the last few years, and we suppose there must be some demand for them. If the student can remember and digest all that appears in this work he will have a very good knowledge of the eye and its diseases.

**A Manual of Infectious Disease.** By E. W. Goodall, M.D., and J. W. Washbourn, C.M.G., M.D. Second Edition. Pp. xii, 426. London: H. K. Lewis. 1908.—The second edition of this excellent manual has been carefully revised and thoroughly brought up to date by Dr. Goodall. Additional chapters on glanders, cerebro-spinal fever and plague, add greatly to its interest and value, and the photographs and new illustrations are well chosen and representative. The book is a most reliable guide to the study of infectious fevers.

**Hygiene and Public Health.** By B. Arthur Whitelegge, C.B., M.D., and George Newman, M.D. Pp. viii, 650. London: Cassell & Company, Ltd. 1908.—This eleventh edition of Whitelegge's well-known manual contains many additional facts and records representing the contribution of the three years since the last edition to the science and practice of public health. While avoiding controversial matter, the information contained is concise and reliable, and the book remains a useful introductory text-book for the student of preventive medicine.

**The Medical Annual.** Bristol: John Wright & Sons, Ltd. 1909.—The twenty-seventh issue of this perennial, appearing early every year, maintains its former characteristics, and by using a thinner paper the publishers have been able to increase the number of its pages without enlarging the volume. Every endeavour has been made to improve and make each volume more useful than its predecessor. Again we have to welcome an excellent series of stereoscopic plates, and an improved stereoscope with which to see them. The double stereoscopic picture of anatomical subjects is so overwhelmingly superior to the simple plane monocular engraving, that no illustrated book of this kind can be satisfactory without this method of demonstration. The stereo view of the larynx, photographed in colours, is a brilliant reproduction.

**Lectures on the Pathology of Cancer.** By CHAS. POWELL WHITE, M.A., M.D. Pp. 83. Manchester: At the University Press. 1908.—Medical literature would have been poorer had these lectures, delivered last summer at the University of Manchester, not been preserved in book form. The subject is the most difficult and the most absorbing in the field of pathology, and the interest that attaches to the publication of the results of cancer research will in a measure be repaid by the study of these lectures. At the outset the author invites criticism by reason of his new classification and nomenclature of tumours. It is difficult to say how far he is justified in this matter. If it can be truly said that an advance in knowledge is thereby attained then it may be forgiven, but whatever the expression of the individual, that of the profession as a whole is conservative to a degree, and time alone will show whether these innovations will rust and decay or become brighter with use. In the microscopical diagnosis of cancers, Dr. Powell White cannot be accused of ultra-progressive tendencies, for he relies upon "infiltration of the surrounding tissues" as the one necessary feature of malignancy. A line of thought suggested by the co-ordinating mechanism of the organism has been followed up, resulting in an interesting chapter. He holds that cancer is a process of infection by cancer cells, cells freed from the co-ordinating influence of the organism. "A cancer," he writes, "cannot be regarded as an individual; it is a colony of cells, and the cells themselves are individuals." In an appendix he briefly summarises his arguments against the advocacy of the parasitic nature of cancer. To bio-chemistry, it would seem, he looks for the evidence upon which will be eventually based that knowledge of tumours and of cancers which, in its complexity and unfathomableness, seems almost akin to the knowledge of life itself. If then the tone be somewhat pessimistic, it must not discourage, but rather stimulate workers to renewed activity. Though one after another come face to face with blank walls and yawning chasms, yet it is certain that someday the walls will be scaled, the chasms bridged, and when that day comes the memory of those who strove and seemed to fail will not be forgotten.

**Handbook for Attendants on the Insane.** Fifth Edition. Pp. xvi, 390. London: Baillière, Tindall & Cox. 1908.—This book, published by the authority of the Medico-Psychological Association, must satisfy a demand, inasmuch as it is now in its thirty-third thousand, the new edition having been made necessary by the extension of the system of training and examination for the certificates of proficiency in nursing the insane, which has been determined by the syllabus of the Education Committee of the Association. The preliminary chapters on anatomy, physiology and hygiene, are followed by an excellent and most useful outline of the accidents to which insane patients are liable, the injuries caused by the violence of others, by suicidal attempts, and other emergencies. A chapter on the general symptomatology of bodily diseases gives much useful and reliable information, but we learn with some dismay that chronic bronchitis is usually caused by the inhalation of tobacco smoke (p. 87). We also notice that no instructions are given as to the disposal of the sputum of phthisical patients. The sections on insanity and its management are, as they should be, the most valuable part of this much-to-be-commended guide.

**Text-Book of Organic Chemistry for Medical Students.** By Dr. G. v. BUNGE. Translated with additions by R. H. ADERS PLIMMER, D.Sc. Pp. viii, 260. London: Longmans, Green and Co., 1907.—So great is the importance of organic chemistry to physiology, pathology and medicine that the medical student is now compelled to have some knowledge of this branch of chemistry, and Professor Bunge, in seventeen lectures, has made a capital selection from the enormous mass of material which has accumulated since 1828. Professor Plimmer's translation is admirable, and the slight modifications he has introduced into the original work are a distinct improvement.

**The Smithsonian Institution: Annual Report for 1907.** Washington: Government Printing Office. 1908.—A paper by Gustave Loisel on the zoological gardens and establishments of Great Britain, Belgium and the Netherlands gives an excellent account of our Bristol Zoological Gardens. In addition to the reports giving an account of the operations for the year, a general appendix comprises a selection of miscellaneous memoirs of interest to collaborators and correspondents of the institution, teachers and others engaged in the promotion of knowledge.

**Notes on Dental Anatomy.** By G. A. PEAKE, M.R.C.S., L.D.S. Pp. viii, 104. London: Claudius Ash, Sons & Co. Ltd. 1908.—This is quite the best book of its kind, and will be found to be of great value in the lecture room and in preparation for examination.

